# AI-enhanced coronary CTA for optimizing surgical planning in CABG: a new frontier in preoperative assessment?

**DOI:** 10.1093/ehjimp/qyag042

**Published:** 2026-03-05

**Authors:** Fatih Kizilyel, Bedirhan Bugra Bayici

**Affiliations:** Department of Cardiovascular Surgery, Kosuyolu High Specialization Education and Research Hospital, Denizer St., No: 2, 34865, Kartal, Istanbul, Turkey

We read with great interest the study by Kero *et al*. evaluating the relationship between artificial intelligence–guided coronary computed tomography angiography (CTA)–derived plaque burden and the severity of myocardial ischemia.^1^ The authors should be congratulated for demonstrating that quantitative plaque metrics—particularly stenosis severity and non-calcified plaque burden—are independently associated with increasing ischemia severity, while also emphasizing the limitations of anatomical assessment alone in predicting functional impairment.

This work is particularly relevant from a cardiac surgical perspective. In patients undergoing coronary artery bypass grafting (CABG), preoperative decision-making has traditionally relied on invasive coronary angiography (CAG). While CAG provides essential information on luminal narrowing and flow dynamics, it offers limited insight into plaque composition, vessel wall pathology, and diffuse atherosclerotic involvement. In contrast, AI-assisted coronary CTA enables objective quantification of plaque burden and distribution, potentially providing a more comprehensive anatomical substrate for surgical planning. CTA should therefore not be viewed merely as a diagnostic tool but also as a critical surgical planning modality.^2^

Importantly, intraoperative reality often differs from angiographic expectations. In daily surgical practice, achieving optimal anastomotic quality in diffusely calcified coronary arteries can be technically challenging. Plaque morphology, calcific burden, and vessel wall integrity frequently determine the feasibility of bypass grafting beyond what is suggested by angiographic stenosis alone.^3^ We frequently encounter diffusely diseased and heavily calcified coronary targets despite only moderate angiographic stenosis. Such discrepancies may lead to intraoperative surprises, impaired distal run-off, and ultimately influence graft strategy and anastomotic quality. In selected patients, the need for coronary endarterectomy becomes evident only intraoperatively; although relatively uncommon, it represents a technically demanding scenario that directly affects operative strategy and patient outcomes.

From this standpoint, the ability of AI-based coronary CTA to characterize plaque distribution and composition may provide clinically meaningful preoperative insights. Anticipating diffusely diseased segments, limited distal run-off, or heavily calcified targets before surgery could facilitate precise operative planning, graft selection, and risk stratification. The authors’ observation regarding the substantial overlap between anatomical plaque metrics and ischemia severity categories further highlight the multifactorial nature of coronary flow limitation. For surgeons, this underscores why graft outcomes cannot always be predicted solely from angiographic stenosis and why an integrated anatomical–functional assessment is essential for optimizing revascularization strategies.^4^

In conclusion, this study provides important evidence that AI-guided coronary CTA offers clinically meaningful information extending beyond initial diagnosis into the domain of surgical planning. Wider adoption and continued refinement of these technologies may reduce intraoperative uncertainty, improve graft strategy, and ultimately enhance the quality and durability of surgical revascularization.^5^ Future studies correlating AI-derived plaque characteristics with intraoperative findings and long-term graft patency would be highly valuable in defining its definitive role in contemporary CABG practice.

## Data Availability

No new data were generated or analyzed in support of this research.

## Lead author biography

Fatih Kizilyel, MD, studied medicine at Istanbul University, Cerrahpaşa Medical Faculty, and earned his cardiovascular surgery degree in 2023 from Siyami Ersek Training and Research Hospital in Istanbul. He currently works as a cardiovascular surgeon and researcher at the Department of Cardiovascular Surgery, Koşuyolu Yüksek İhtisas Training and Research Hospital in Istanbul. His clinical practice primarily focuses on coronary artery bypass surgery, valvular heart disease, and myocardial protection. His academic and research interests center on improving surgical outcomes through evidence-based approaches, clinical data analysis, and the integration of artificial intelligence into cardiovascular surgery. Dr. Kizilyel is particularly focused on the application of AI models in preoperative surgical planning, heart team decision-making, and risk stratification for complex open-heart procedures.
